# Citation clarification regarding feline eltrombopag in the “ACVIM consensus statement on the treatment of immune thrombocytopenia in dogs and cats”

**DOI:** 10.1093/jvimsj/aalag213

**Published:** 2026-09-11

**Authors:** Jihun Kim, Dongmi Kwak

**Affiliations:** College of Veterinary Medicine, Kyungpook National University, Daegu 41566, Korea; College of Veterinary Medicine, Kyungpook National University, Daegu 41566, Korea; Institute for Veterinary Biomedical Science, College of Veterinary Medicine, Kyungpook National University, Daegu 41566, Korea

Dear Editor,

We read with great interest the American College of Veterinary Internal Medicine consensus statement on the treatment of immune thrombocytopenia (ITP) in dogs and cats.^1^ The statement provides an important and highly valuable framework for evidence-based management of veterinary ITP. While reviewing the literature cited in the section on thrombopoietin receptor agonists in cats, we identified a specific citation that warrants clarification. Although this issue does not affect the principal recommendations of the consensus statement, this clarification may help readers more accurately trace the experimental evidence supporting the cited statement.

In the treatment consensus statement, the evidence summary for thrombopoietin receptor agonists in cats states, “Eltrombopag is unlikely to be effective in cats,” citing reference 93.^1^ Reference 93 corresponds to the study by Erickson-Miller et al.^2^ describing the discovery and characterization of the nonpeptidyl thrombopoietin receptor agonist SB 394725. Although that study provides relevant evidence regarding the species specificity of this class of small-molecule thrombopoietin receptor agonists, the compound evaluated was SB 394725, which is a different compound from eltrombopag (SB-497115).

Direct feline experimental evidence for SB-497115 appears instead in an earlier conference abstract by Erickson-Miller et al.^3^ In that study, SB-497115-induced signaling was detected in human and chimpanzee platelets, but not in feline platelets.^3^ SB-497115 was subsequently developed clinically as eltrombopag.^4^ Therefore, the conclusion in the consensus statement that eltrombopag is unlikely to be effective in cats is entirely consistent with the available experimental evidence; however, the cited reference does not evaluate the specific compound named in the statement. We respectfully suggest that the citation accompanying this statement be replaced with, or supplemented by, the earlier report containing direct feline data for SB-497115. This clarification would allow readers to more directly trace the experimental evidence underlying this feline-specific conclusion.

## Abbreviations

ACVIM: American College of Veterinary Internal Medicine
ITP: immune thrombocytopenia
